# Acute Mitral Valve Regurgitation Presenting With Right Upper Lobe Opacification

**DOI:** 10.7759/cureus.29078

**Published:** 2022-09-12

**Authors:** John Wallis, Daniel I Shpigel, Dane O'Donnell, Meryl Ponce, Mark J Decaro

**Affiliations:** 1 Department of Medicine, Division of Cardiology, Stony Brook University Hospital, Stony Brook, USA; 2 Department of Emergency Medicine, Thomas Jefferson University Hospital, Philadelphia, USA; 3 Department of Emergency Medicine, Doctors Community Hospital, Lanham, USA; 4 Department of Medicine, Division of Cardiology, Thomas Jefferson University Hospital, Philadelphia, USA

**Keywords:** mitral valve regurgitation, acute mitral regurgitation, mitral regurgitation, cardiogenic shock, non-ischemic cardiac pathologies, papillary muscle rupture, acute cardiogenic pulmonary edema, opacification of hemithorax, acute mitral valve regurgitation, mitral valve replacement

## Abstract

There is literature describing unilateral or focal pulmonary edema due to mitral regurgitation. The proposed mechanism is a regurgitant jet propelling blood towards the orifice of a particular pulmonary vein within the left atrium, which selectively pressurizes that vein. The increased hydrostatic pressure is transmitted to the pulmonary capillaries that drain into that vein, causing focal consolidation. A 62-year-old female presented with acute hypoxic respiratory failure. Her dyspnea started suddenly and she was unresponsive when she arrived at the emergency department via emergency medical services. Her initial oxygen saturation was 23% and she was immediately intubated. Sequential chest radiographs demonstrated dense consolidation in the right upper lung field and then opacification of the right hemithorax. These asymmetric lung findings were suspicious for infectious etiology but she was afebrile with no respiratory secretions and had normal inflammatory markers. Echocardiography showed a ruptured anterior papillary muscle causing a flail mitral valve leaflet with severe mitral regurgitation. The patient developed cardiogenic shock; she had an intra-aortic balloon pump placed for afterload reduction and was taken to the operating room for an emergency mitral valve replacement. Her clinical status rapidly improved and she made a full recovery. As in this case, acute mitral regurgitation can present with sudden life-threatening respiratory failure and cardiogenic shock so prompt diagnosis is critical. This is often misdiagnosed as pneumonia or other respiratory illnesses. Awareness, early diagnosis, and treatment of this entity could provide significant morbidity and mortality benefits for patients.

## Introduction

Shortness of breath is a common chief complaint seen in the emergency department and carries a broad differential diagnosis. Chest radiography, a valuable and commonly used tool, can help clinicians narrow differential diagnoses and provide appropriate patient care. Unilateral infiltrates on chest radiography, often seen in bacterial pneumonia, may evoke an infectious etiology of dyspnea. However, focal or unilateral pulmonary edema has been described in patients with mitral regurgitation from numerous causes, including spontaneous valve perforation [1], valve perforation due to infectious endocarditis [2], transient papillary muscle dysfunction due to myocardial ischemia [3], and spontaneous papillary muscle or chordae tendineae rupture [4-6]. Case reports show that this finding is often initially mistaken for pneumonia or other respiratory illnesses [7]. Diagnostic anchoring based on unilateral radiographic consolidation has the potential to cause significant morbidity and mortality in similar cases, highlighting the importance of a high index of suspicion, early diagnosis, and proper management [8].

## Case presentation

A 62-year-old female presented to the emergency department with acute respiratory failure. Her past medical history included obstructive sleep apnea with the use of bilevel positive airway pressure at night, obesity hypoventilation syndrome, systemic hypertension, pulmonary hypertension, mild to moderate mitral regurgitation complicated by pulmonary edema, asthma, and the use of home oxygen (two liters via nasal cannula). A transthoracic echocardiogram performed five months before the presentation during hospitalization for dyspnea showed normal biventricular size and function, mild left atrial dilation, normal mitral valve structure with mild to moderate regurgitation, and a mildly elevated pulmonary artery systolic pressure. A chest radiograph performed one month before the presentation showed no evidence of pulmonary edema. Her home medications included furosemide, losartan, inhaled fluticasone and vilanterol, promethazine syrup, nebulized albuterol, and benzonatate.

Per the patient’s husband, she was in her normal state of health until awakening in the morning and feeling short of breath after using the restroom; the shortness of breath was refractory to albuterol nebulizer treatments self-administered at home for a presumed asthma exacerbation. At that time, she appeared to have labored breathing, prompting the patient and her husband to call emergency medical services (EMS). In the ambulance, she was initially alert and awake with a normal mental status but suddenly became unresponsive; her oxygen saturation dropped to 20% and her heart rate increased to 160 beats per minute. She arrived at the emergency department via EMS, unresponsive, with an oxygen saturation of 23% and sinus tachycardia; initial examination revealed diffuse rhonchi and a Glasgow Coma Scale (GCS) score of six. She was immediately intubated; she required high ventilator settings (fraction of inspired oxygen (FiO2) at 100%, positive end-expiratory pressure (PEEP) at 16 mbar), and neuromuscular blockade to achieve adequate oxygenation.

**Figure 1:**
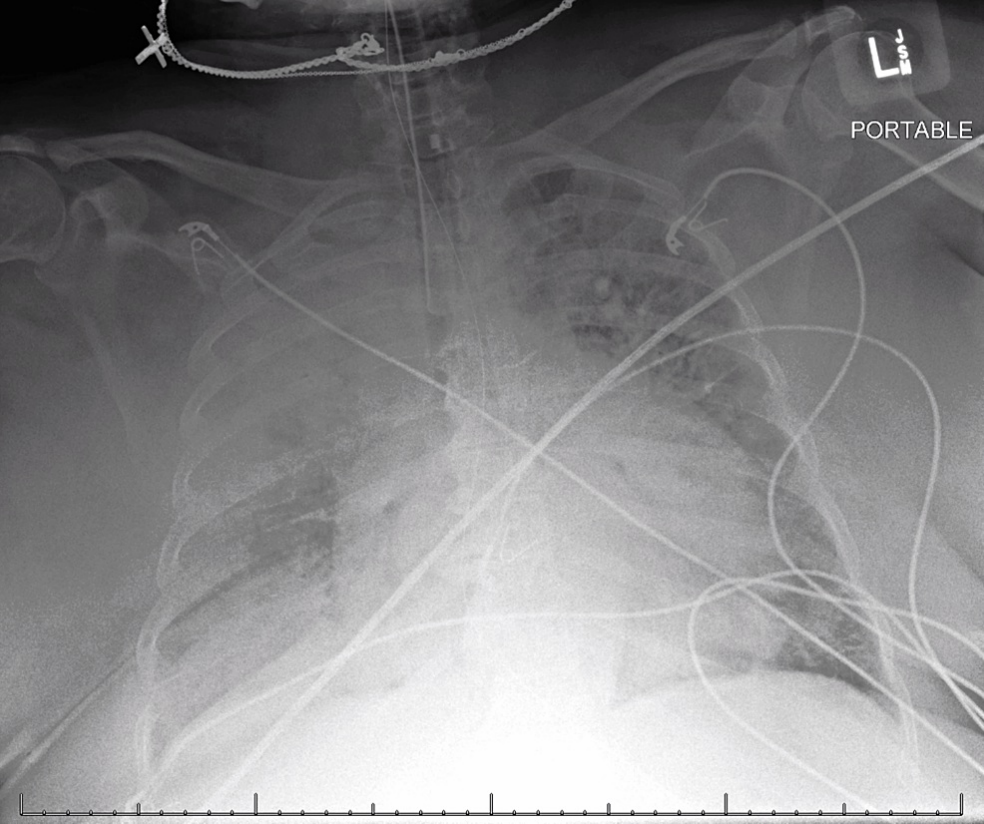
Following intubation, the first portable chest radiograph shows mild cardiomegaly, background interstitial pulmonary edema, dense consolidation in the right upper lobe, and patchy opacity at the right lung base.

Initial chest radiography demonstrated mild cardiomegaly, background interstitial pulmonary edema, and a dense consolidation in the right upper lobe with a patchy opacity at the right lung base. (Figure 1).

**Figure 2:**
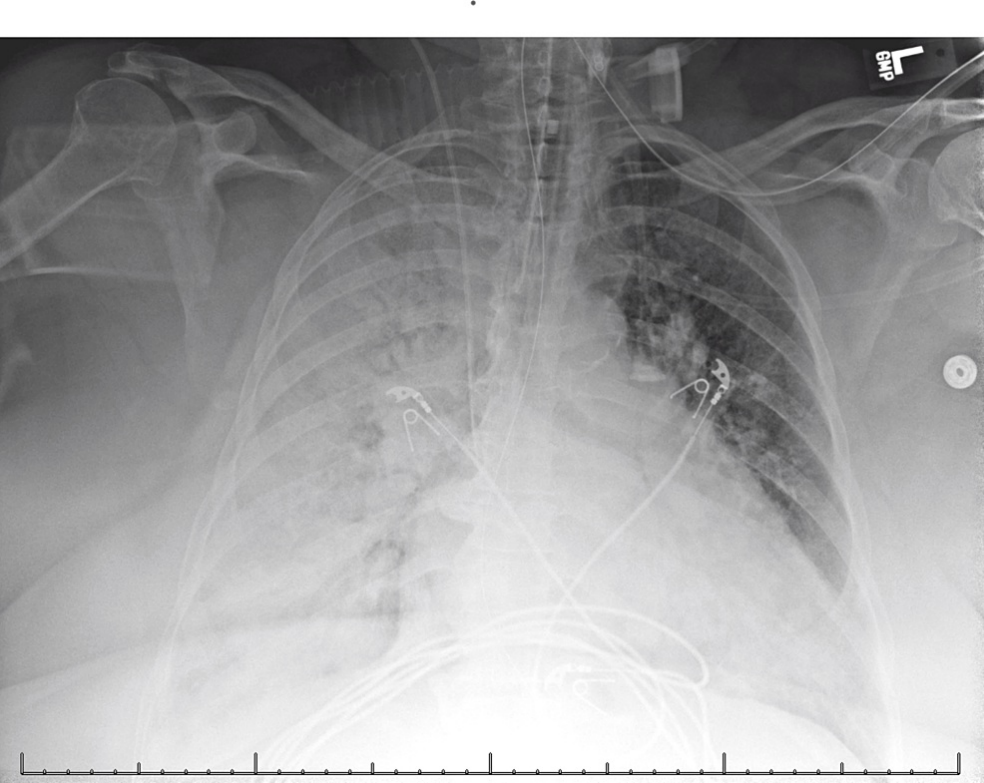
Subsequent chest radiograph showing nearly complete opacification of the right hemithorax and progressive opacification of the left lung base.

A subsequent chest radiograph showed nearly complete opacification of the right hemithorax and progressive opacification of the left lung base (Figure 2).

These asymmetric lung findings were suspicious for an infectious etiology, including coronavirus disease 2019 (COVID-19) and bacterial pneumonia. She tested negative for COVID-19 (sample taken from tracheal aspiration). She had a mild leukocytosis without neutrophilic predominance, and inflammatory markers were not consistent with severe infection (Tables 1, 2). Given these objective findings, the rapid progression of respiratory signs and symptoms, normothermia, a lack of respiratory secretions, and cardiac etiologies were investigated.

Her initial ECG showed sinus tachycardia at 132 beats per minute with no ischemic changes. She was treated for acute coronary syndrome with aspirin, atorvastatin, and continuous unfractionated heparin, although her troponin elevation was most likely a result of profound hypoxia rather than coronary thrombosis. This treatment was initiated before successive troponin results, which were inconsistent with acute coronary syndrome. The third troponin result acquired was the peak value (Table 2).

**Table 1:** Hematological parameters

Parameter	Laboratory value	Reference range
Hemoglobin	11.4 g/dL	12.5-15.0 g/dL
White Blood Cell count	12.4 B/L	4.0-11.0 B/L
Hematocrit	39.7%	36.0-46.0%
Platelet count	299 B/L	140-400 B/L
Neutrophils relative	52.0%	40.0-73.0%
Lymphocytes relative	36.3%	20.0-44.0%
Monocytes relative	8.8%	3.0-13.0%
Eosinophils relative	1.3%	0.0-6.0%

**Table 2:** Biochemical parameters

Parameter	Laboratory value	Reference range
Sodium	137 mmol/L	135-146 mmol/L
Potassium	4.4 mmol/L	3.3-4.8 mmol/L
Bicarbonate	27 mmol/L	24-32 mmol/L
Anion gap	12 mmol/L	4-16 mmol/L
Glucose	307 mg/dL	70-100 mg/dL
Total Bilirubin	0.4 mg/dL	0.1-0.9 mg/dL
Aspartate transaminase (AST )/ Alanine transaminase (ALT)	23/20 IU/L	7-35/<30 IU/L
Lactate	3.2 mmol/L	0.5-2.0 mmol/L
High-sensitivity troponin (7:38am)	66 ng/L	<19 ng/L
High-sensitivity troponin (10:20am)	144 ng/L	<19 ng/L
High-sensitivity troponin (2:04pm)	151 ng/L	<19 ng/L
Procalcitonin	0.05 ng/mL	<0.08 ng/mL
Pro-B type natriuretic peptide	246 pg/mL	<125 pg/mL
C-reactive protein	0.90 mg/dL	<0.80 mg/dL

**Figure 3:**
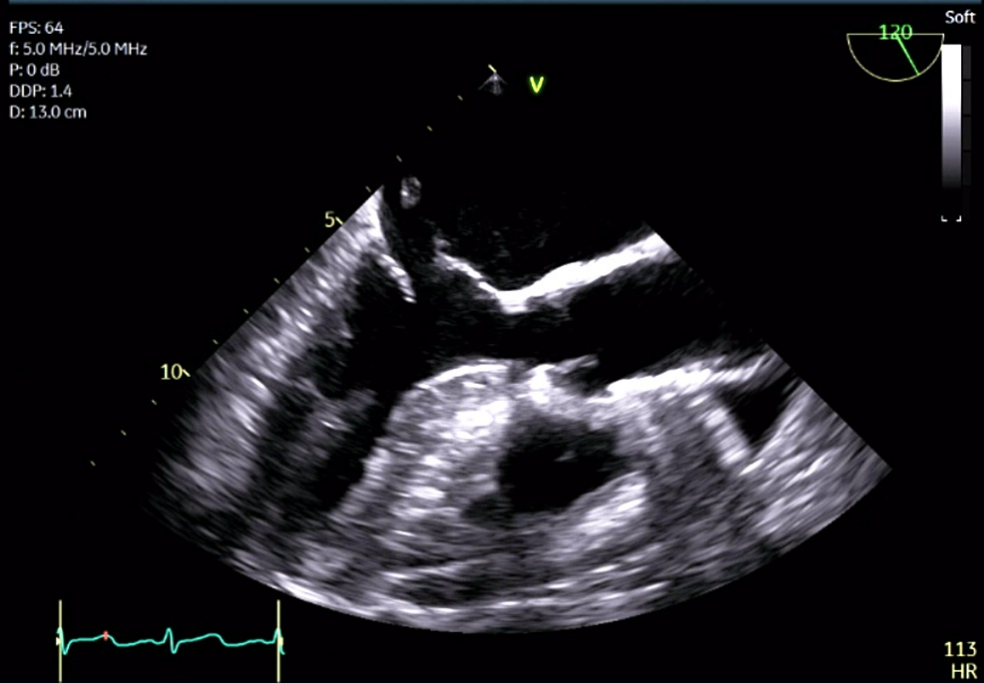
Transesophageal echocardiogram showing flail anterior mitral valve leaflet during systole with the ruptured anterior papillary muscle attached to the chordae tendineae.

A transthoracic echocardiogram showed normal biventricular function but detected a flail anterior mitral valve leaflet with significant mitral regurgitation and an eccentric jet. The patient became hypotensive and required vasopressors, her extremities became poikilothermic, and she was anuric despite diuretics, raising concern for cardiogenic shock. The patient was emergently transferred to a tertiary medical center where a left heart catheterization demonstrated minimal luminal irregularities in all vessels and an intra-aortic balloon pump was placed for afterload reduction. A transesophageal echocardiogram (TEE) confirmed severe mitral regurgitation and showed a ruptured anterior papillary muscle (Figure 3).

**Figure 4:**
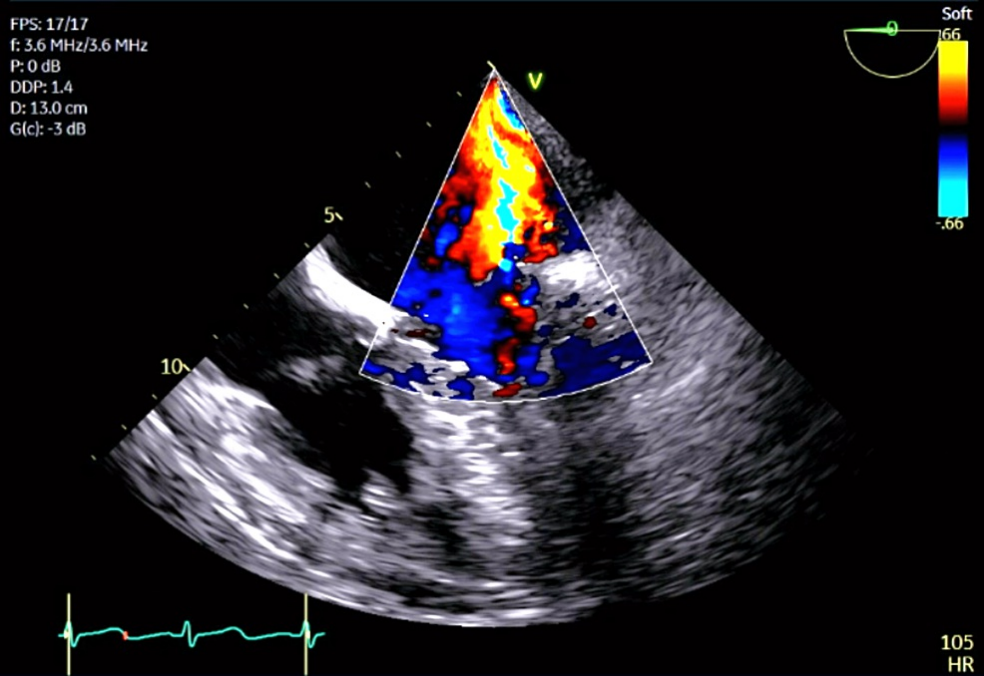
Transesophageal echocardiogram in color Doppler mode demonstrating mitral valve regurgitation.

**Figure 5:**
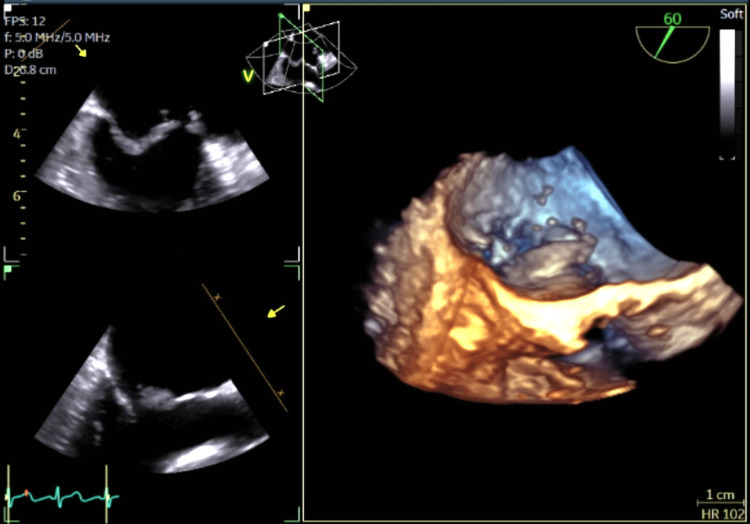
Flail mitral valve orifice demonstrated by 3D post-processing of transesophageal echocardiogram.

The TEE showed severe systolic flow reversal in the right upper pulmonary vein and only mild systolic flow reversal in the other three pulmonary veins. Figure 4 and Figure 5 show, respectively, mitral valve regurgitation on TEE in color Doppler mode and the mitral valve flail orifice after 3D post-processing.

**Figure 6:**
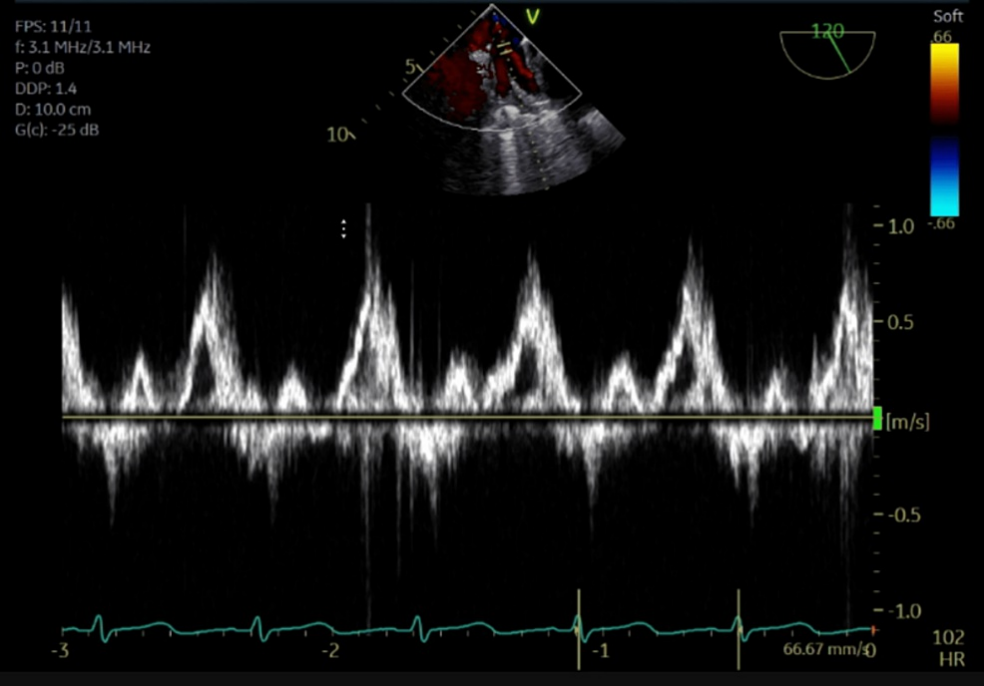
Pulsed-wave Doppler of the left superior pulmonary vein showing systolic flow reversal.

**Figure 7:**
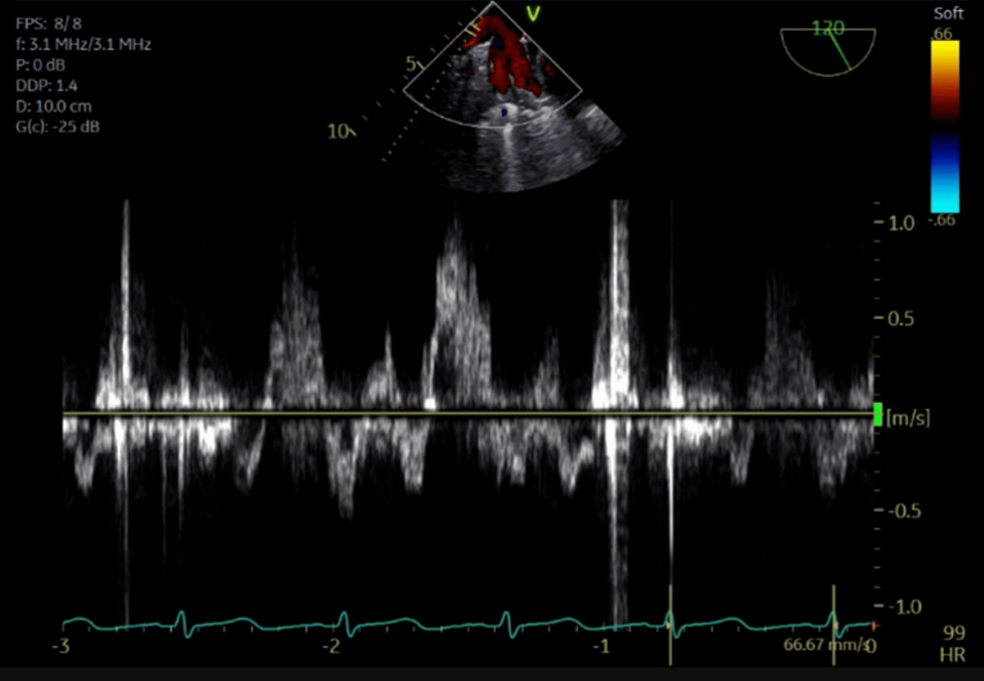
Pulsed-wave Doppler of the left inferior pulmonary vein showing systolic flow reversal.

**Figure 8:**
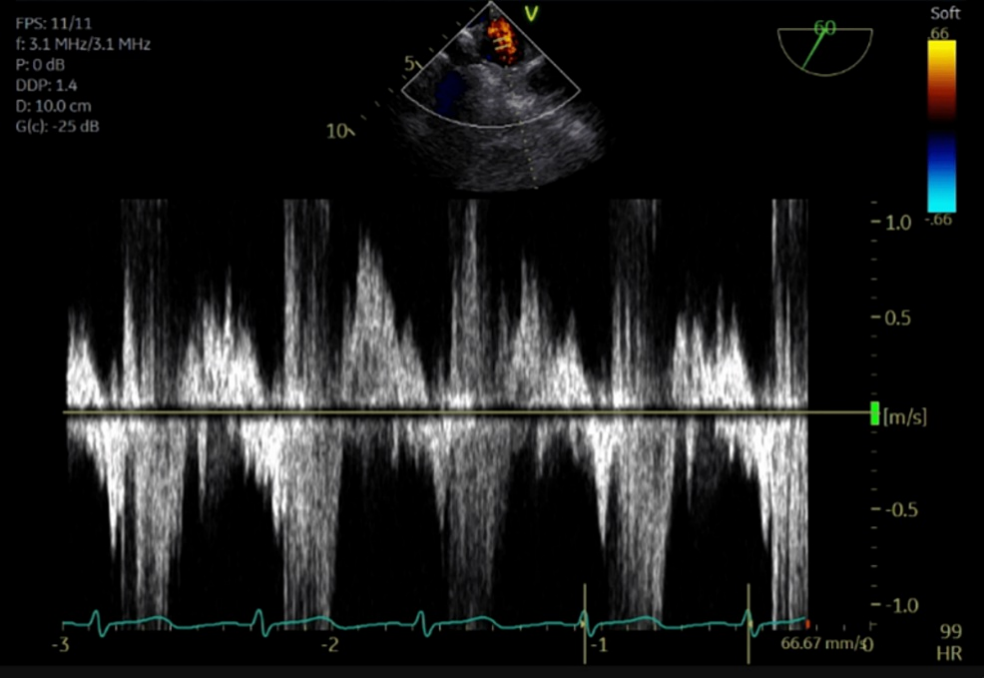
Pulsed-wave Doppler of the right superior pulmonary vein showing systolic flow reversal.

**Figure 9:**
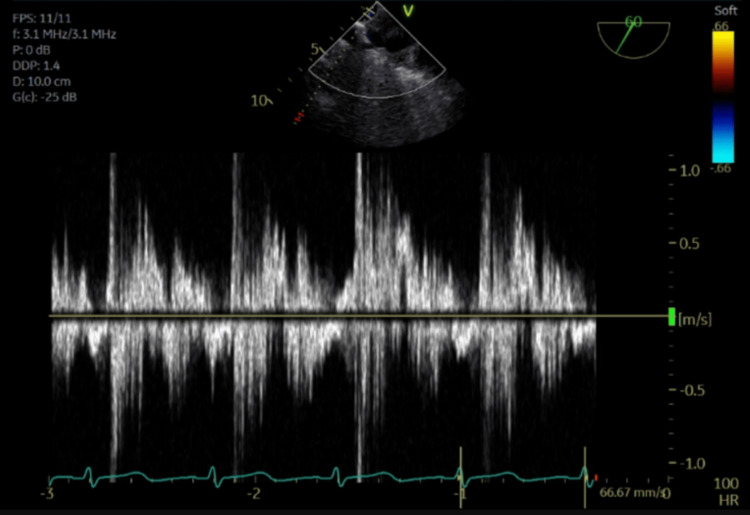
Pulsed-wave Doppler of the right inferior pulmonary vein showing systolic flow reversal.

The pulsed-wave Doppler images demonstrate pulmonary vein flow reversal, noting the highest degree of flow reversal in the right superior pulmonary vein (Figures 6-9).

**Figure 10:**
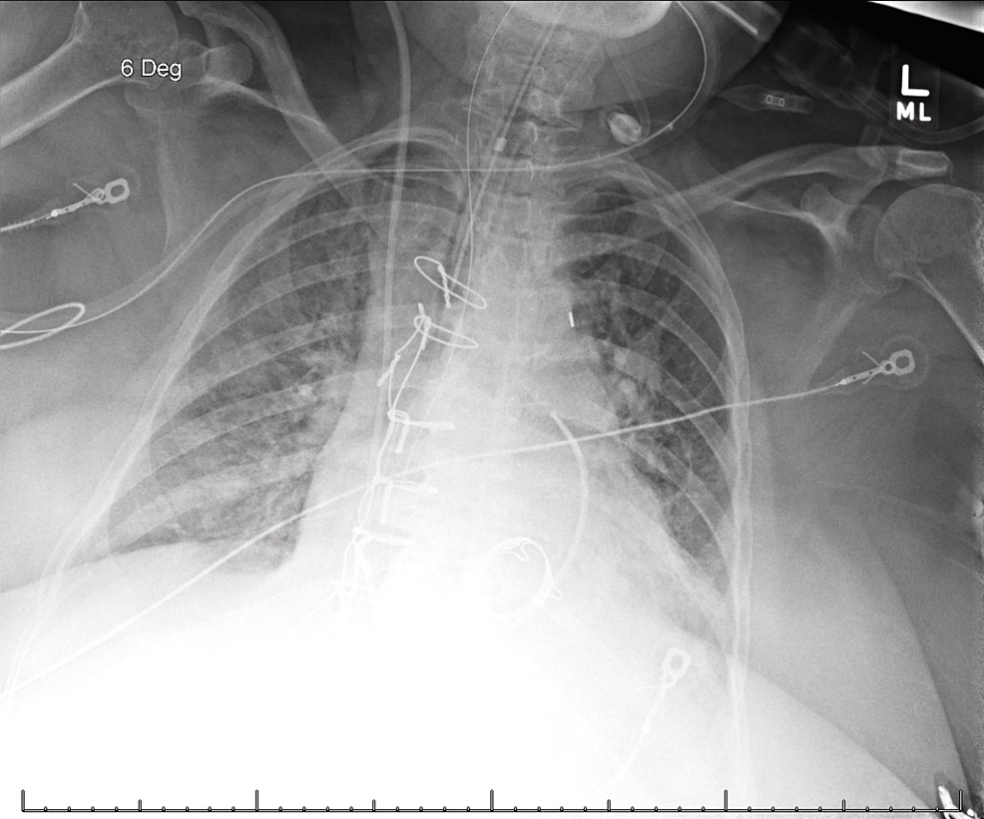
Post-operative chest radiograph (approximately 36 hours after presentation) showing significant improvement in right-sided infiltrates after mitral valve replacement.

She was taken to the operating room for emergent bioprosthetic mitral valve replacement. Afterward, her clinical status improved; she began to void, her pulmonary artery pressures were corrected, and she was extubated 72 hours after the presentation. Subsequent chest radiography showed rapid resolution of the asymmetric infiltrates (Figure 10).

## Discussion

This was a dramatic presentation of spontaneous, non-ischemic papillary muscle rupture, causing severe acute mitral regurgitation and respiratory failure from the resulting flash pulmonary edema. The patient was critically ill within one to two hours of symptom onset. Of particular interest is the initial chest radiograph showing asymmetric opacification with dense consolidation of the right upper lobe. This appearance was initially concerning for an infectious etiology, which could have delayed the diagnosis.

Focal or unilateral pulmonary edema has been described in patients with mitral regurgitation from numerous causes, including spontaneous valve perforation [1], valve perforation due to infectious endocarditis [2], transient papillary muscle dysfunction due to myocardial ischemia [3], and spontaneous papillary muscle or chordae tendineae rupture [4-6]. Case reports show that this finding is often initially mistaken for pneumonia or other respiratory illnesses [7].

Schnyder et al. found that 9% of patients with severe mitral regurgitation had chest radiography findings that were "localized or predominant" in the right upper lobe [8]. Attias et al. studied 869 patients admitted with cardiogenic pulmonary edema and found that 2.1% had unilateral pulmonary edema (UPE). Notably, all patients with UPE were found to have severe mitral regurgitation. Of patients with cardiogenic pulmonary edema and severe mitral regurgitation, 75% had bilateral findings and 25% had unilateral findings. Only 6% of patients with bilateral pulmonary edema received antibiotics, whereas 61% of patients with unilateral pulmonary edema received antibiotics. This shows that focal chest radiograph findings often invoke an infectious etiology among clinicians. The outcomes vary greatly depending on the initial imaging findings. Patients with UPE were found to have significantly lower blood pressure on presentation, higher use of noninvasive or invasive positive pressure ventilation, and more frequent use of vasopressors or inotropes. Patients with severe mitral regurgitation had in-hospital mortality of 39% when presenting with unilateral findings compared to 6% when presenting with bilateral findings. Therefore, presentation with focal radiograph findings not only delays the diagnosis but has been shown to correlate with worse morbidity and mortality [9].

There is a proposed mechanism for localized pulmonary edema due to mitral regurgitation. Typically, systolic or diastolic left ventricular failure leads to increased pressure within the left atrium, which is transmitted symmetrically to each of the pulmonary veins. This leads to increased hydrostatic pressure within pulmonary capillaries, causing a uniform degree of pulmonary edema throughout the lungs. It is thought that asymmetric pulmonary edema is due to a mitral regurgitant jet that propels blood selectively towards the orifice of a particular pulmonary vein within the left atrium. If a regurgitant jet causes increased pressure within that pulmonary vein, it would transmit increased hydrostatic pressure selectively to the pulmonary capillaries that drain into that pulmonary vein, causing focal edema.

This mechanism is supported by Kashiura et al., who described two cases of unilateral pulmonary edema from severe acute mitral regurgitation. The first case involved an eccentric jet blowing towards the right side of the left atrium and a patient who presented with right-sided opacities. The second case involved a jet blowing towards the left side of the left atrium and a patient who presented with left-sided opacities [2]. In this case, a regurgitant jet was directed towards the right upper pulmonary vein, causing right upper lung opacification, substantiated by TEE pulse wave doppler images confirming severe systolic flow reversal within the right upper pulmonary vein.

## Conclusions

As in this case, acute mitral regurgitation can present with sudden life-threatening respiratory failure and cardiogenic shock, so prompt diagnosis is critical. This is often misdiagnosed as pneumonia or other respiratory illnesses. Patients with unilateral lung opacification due to mitral regurgitation have delays in diagnosis and worse outcomes compared to similar patients with bilateral pulmonary edema. In this case, the key to the diagnosis was the reported history of sudden-onset dyspnea that rapidly progressed to respiratory failure.

In conclusion, acute mitral regurgitation should be considered in the differential diagnosis for patients with focal pulmonary opacities or complete opacification of a hemithorax in the appropriate clinical context, such as sudden-onset hypoxic respiratory failure, especially if vitals, examination, and laboratory biomarkers are inconsistent with severe infection. Awareness, early diagnosis, and treatment of this entity could provide significant morbidity and mortality benefits for patients. In this case, the findings support the previously proposed mechanism of a regurgitant jet selectively pressurizing a pulmonary vein, leading to focal consolidation.
